# Pregnancy Discovered During Admission for Psychotic Relapse: A Case Study on Diagnostic Overshadowing and Physical Health Screening

**DOI:** 10.1192/bjo.2026.11172

**Published:** 2026-06-30

**Authors:** Sophie Nocton, Lian Chua, Elizabeth Nocton

**Affiliations:** 1 Leeds Community Healthcare NHS Trust, Leeds, United Kingdom; 2 Bradford District Care NHS Foundation Trust, Bradford, United Kingdom; 3 The University of Manchester, Manchester, United Kingdom

## Abstract

**Aims:**

People with severe mental illness experience significant physical health morbidity and are vulnerable to diagnostic overshadowing. This may be particularly relevant where reproductive health factors overlap with psychiatric symptoms. This case study describes an unrecognised pregnancy identified during admission for psychotic relapse in a woman with schizoaffective disorder and explores learning points for inpatient, community and antenatal care. The hypothesis underpinning this work is that routine pregnancy testing and proactive consideration of reproductive health in women of childbearing potential may reduce the risk of delayed pregnancy recognition and associated harm.

**Methods:**

A 35-year-old woman with schizoaffective disorder was managed in the community on a Community Treatment Order with depot antipsychotic medication. She developed persecutory beliefs that her food was being poisoned, reported altered taste and smell, alongside nausea and vomiting, and gradually reduced her food intake, leading to weight loss and non-attendance for depot medication. Due to increasing self-neglect and psychotic relapse, she was recalled to hospital under the Mental Health Act. During admission, antipsychotic treatment was recommenced and partial insight returned. As part of routine physical health monitoring, she was noted to weigh under 50 kg. Medical review identified an unexplained abdominal mass, prompting urgent ultrasound, which demonstrated a previously unrecognised 27-week pregnancy.

**Results:**

Retrospective reflection suggested that first-trimester pregnancy symptoms may have interacted with psychotic experiences. Symptoms consistent with hyperosmia and hypergeusia, recognised features of early pregnancy, may have contributed to altered taste perception and heightened sensitivity to food. Nausea and vomiting associated with morning sickness may have further reinforced persecutory beliefs that food was being poisoned. This cluster of physical symptoms appeared to coincide with increasing fearfulness of professionals, medication non-concordance and subsequent psychotic relapse, highlighting the complex, bidirectional relationship between physical health symptoms and psychopathology. The pregnancy was identified incidentally rather than through routine screening, illustrating how pregnancy-related physiological changes may be misattributed to psychiatric illness in both community and inpatient settings.

**Conclusion:**

This case illustrates the risk of diagnostic overshadowing at the interface of physical and mental health in severe mental illness. It highlights the importance of routine pregnancy testing for women of childbearing potential on admission and maintaining clinical curiosity when physical changes accompany psychiatric deterioration. In community settings, emerging physical symptoms, weight change or non-attendance should prompt consideration of pregnancy and clearer screening pathways. While individual circumstances vary, proactive physical health screening may support safer, more holistic psychiatric and antenatal care.

